# Secretion and uptake of copper via a small copper carrier in blood fluid

**DOI:** 10.1093/mtomcs/mfac006

**Published:** 2022-02-24

**Authors:** B D Gioilli, T Z Kidane, H Fieten, M Tellez, M Dalphin, A Nguyen, K Nguyen, M C Linder

**Affiliations:** Department of Chemistry and Biochemistry, California State University, 800 N State College Blvd., Fullerton, CA 92834-6866, USA; Department of Chemistry and Biochemistry, California State University, 800 N State College Blvd., Fullerton, CA 92834-6866, USA; Department of Clinical Sciences of Companion Animals, Faculty of Veterinary Medicine, Utrecht University, 3584 CM Utrecht, the Netherlands; Department of Chemistry and Biochemistry, California State University, 800 N State College Blvd., Fullerton, CA 92834-6866, USA; Department of Chemistry and Biochemistry, California State University, 800 N State College Blvd., Fullerton, CA 92834-6866, USA; Department of Chemistry and Biochemistry, California State University, 800 N State College Blvd., Fullerton, CA 92834-6866, USA; Department of Chemistry and Biochemistry, California State University, 800 N State College Blvd., Fullerton, CA 92834-6866, USA; Department of Chemistry and Biochemistry, California State University, 800 N State College Blvd., Fullerton, CA 92834-6866, USA

**Keywords:** copper carrier, secretion, uptake, blood, urine

## Abstract

Studies with Wilson disease model mice that accumulate excessive copper, due to a dysfunctional ATP7B “copper pump” resulting in decreased biliary excretion, showed that the compensatory increase in urinary copper loss was due to a small copper carrier (∼1 kDa) (SCC). We show here that SCC is also present in the blood plasma of normal and Wilson disease model mice and dogs, as determined by ultrafiltration and size exclusion chromatography (SEC). It is secreted by cultured hepatic and enterocytic cells, as determined by pretreatment with ^67^Cu nitrilotriacetate (NTA) or nonradioactive 5–10 μM Cu-NTA, and collecting and examining 3 kDa ultrafiltrates of the conditioned media, where a single major copper peak is detected by SEC. Four different cultured cell types exposed to the radiolabeled SCC all took up the ^67^Cu at various rates. Rates differed somewhat when uptake was from Cu-NTA. Uptake of SCC-^67^Cu was inhibited by excess nonradioactive Cu(I) or Ag(I) ions, suggesting competition for uptake by copper transporter 1 (CTR1). Knockout of CTR1 in fibroblasts reduced uptake rates by 60%, confirming its participation, but also involvement of other transporters. Inhibitors of endocytosis, or an excess of metal ions taken up by divalent metal transporter 1, did not decrease SCC-^67^Cu uptake. The results imply that SCC may play a significant role in copper transport and homeostasis, transferring copper particularly from the liver (but also intestinal cells) to other cells within the mammalian organism, as well as spilling excess into the urine in copper overload—as an alternative means of copper excretion.

## Graphical Abstract

**Graphical Abstract:**
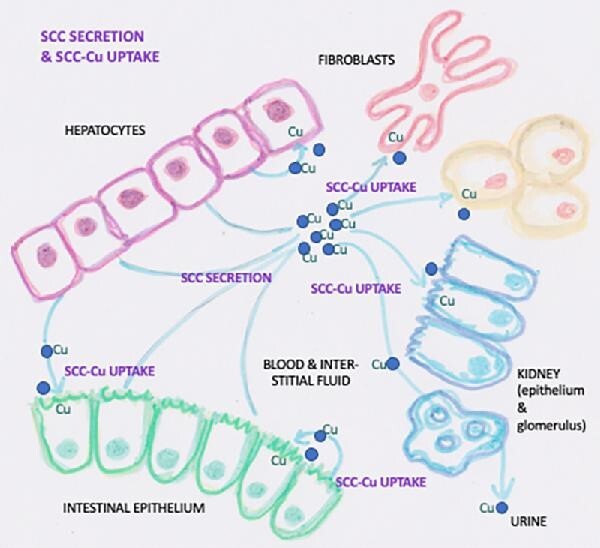
Small copper carrier (blue dots) is secreted into blood by hepatic and intestinal cells, and taken up by the same cells and others.

## Introduction

Copper is a fundamentally important trace element in all living organisms, with a broad variety of functions ranging from support of oxidative phosphorylation (complex IV of the electron transport chain), antioxidant activity (Cu/Zn superoxide dismutases, ceruloplasmin), and formation of certain hormones (dopamine monooxygenase, peptidylglycine alpha-hydroxylating monooxygenase), to melanin production, maturation of collagen and elastin (lysyl oxidase), angiogenesis, and others.^1–8^ Copper is supplied to various cells and tissues in support of these functions via carriers, primarily proteins, that flow through the circulation and interstitial spaces, maintaining a remarkably constant level of this metal in the various compartments. This constancy of tissue copper concentrations is maintained not just within a given mammalian species but even between species.^1,4,8^ Although a great deal is known about the mechanisms involved in maintaining copper homeostasis, a great deal more is yet to be explained and discovered. In human adults, the diet provides about 1 mg Cu per day, but several additional milligrams of Cu flow into the digestive tract as part of saliva, bile, and other gastrointestinal juices.^4,8^ Most but not all of that Cu is reabsorbed—along with most dietary copper.^8^ With such high levels of Cu recycling in and out of the digestive tract, homeostasis is mainly regulated by adjusting copper excretion rather than absorption.

The main route of Cu excretion is the bile produced by hepatocytes. Indeed, the liver plays a central role in Cu homeostasis by pumping excess Cu into the bile to prevent accumulation, and otherwise adjusting this output depending on the amounts of Cu needed for liver cells and those in other tissues. The liver also continuously transfers a significant portion of the Cu absorbed by the intestine into the blood. This is mainly in the form of the most abundant Cu protein in blood plasma, ceruloplasmin, which has several different functions, one of which is to distribute the metal to cells all over the organism.^7–9^

Excretion of Cu into the bile requires the activity of one of two ATP-dependent Cu “pumps” (ATP7B) in the *trans*-Golgi network (TGN). For Cu transfer to the bile, ATP7B migrates/traffics to the apical portions of the hepatocyte plasma membrane that forms bile canaliculi.^8,10,11^ If ATP7B is dysfunctional or poorly expressed, Cu accumulates in hepatocytes, eventually leading to lethal damage to the liver and some other organs, which is termed Wilson disease (a not-uncommon genetic disorder).^12,13^ Expression of ATP7B is relatively limited among cell types, being particularly important in hepatocytes, with small amounts also expressed in cells of some other tissues, including the brain, and more significant amounts in the lactating mammary gland.^14,15^ Both liver and lactating mammary gland cells produce ceruloplasmin (for blood and milk, respectively),^1–4,14^ and the Cu needed for ceruloplasmin is provided to the apo form of the protein in the TGN also by ATP7B, prior to exocytosis within vesicles that fuse with the basolateral plasma membrane leading to the blood.^8,11,12^ A similar but not identical Cu pump (ATP7A), also found in the TGN and which also traffics to the cell plasma membrane, is thought to have a different role, namely that of releasing Cu into the blood.^3,12,15^ This has been particularly important for intestinal Cu absorption, where it has been amply demonstrated that a knockout or dysfunction of ATP7A results in severe Cu deficiency, known as Menkes disease.^3,16^ How important this export-into-blood activity of ATP7A is for Cu transport and homeostasis in other parts of the mammalian organism is still somewhat unclear.^8^ ATP7A is present in hepatocytes but only in low amounts^8,17^ but may normally provide an additional means of releasing hepatocyte Cu into the blood. Lactating mammary epithelial cells express both ATP7A and B in more equal amounts.^15^ In lactation, expression of ATP7A can modulate cellular Cu concentrations in mammary epithelial cells, perhaps to prevent an excess going into the milk, since overexpression of ATP7A in mammary epithelial cells results not only in lower levels of cellular Cu but a decreased secretion of Cu into milk and milk ceruloplasmin.^15^

As concerns uptake of Cu from the gut and from body fluids and blood, only one specific uptake transporter (copper transporter 1, CTR1) has as yet been identified,^18,19^ although others clearly exist.^20–22^ CTR1 has a trimeric configuration, with a central pore through which Cu(I) ions are transferred via methionine and cysteine residues.^23–25^ Silver ions [Ag(I)]—also absorbed by CTR1—competitively inhibit uptake of copper by this transporter.^26,27^ Since it has been shown in many cell types that a large proportion of Cu(I) uptake is not inhibited by excess Ag(I),^21,22^ Cu is also being absorbed by other transporters or processes. A chloride-dependent uptake mechanism has also been identified in the case of enterocytic cells,^28^ and some evidence has implicated divalent metal transporter 1 (DMT1) in Cu uptake in certain conditions,^29–31^ although there also is strong evidence to the contrary.^32,33^ Other than in the gut, Cu is delivered to the uptake transporters especially by the plasma proteins albumin, ceruloplasmin, and transcuprein/alpha-2-macroglobulin, as has been demonstrated for a variety of cultured cells.^9,21,22^

Animal models of Wilson disease have been identified or developed, particularly in rodents but also in dogs.^34,35^ A mouse model, produced by knocking out Atp7b, has been available for studying Cu transport and metabolism for some time.^36,37^ These mice accumulate large amounts of Cu in the liver (and some other tissues), and as in humans with Wilson disease, their urinary Cu excretion also markedly rises.^38^ Normally in mammals, only a small fraction of daily Cu excretion is via the urine,^1,4,8^ and so it was of interest to further investigate urinary Cu excretion as an alternative to that of biliary Cu that might be activated in this disease. This was examined by Gray and others in the Lutsenko lab, using the Atp7b−/− mice, which showed (as already indicated) that large amounts of excess Cu accumulated in the liver, and urinary Cu concentrations greatly increased.^38^ They also showed that, consistent with lack of Atp7b and using radioactive Cu, turnover of liver Cu was markedly decreased, indicating that most of the incoming Cu was stuck in the liver. Gray *et al.* then went on to investigate the nature of the extra Cu appearing in the urine of the Atp7b−/− mice. They found that it was associated with a small molecule, considerably larger than Cu-diHis or Cu-Gly-His-Lys, but <3 kDa, and they designated it as a ‘small copper carrier’ (SCC). It so happened that at about the same time, Hille Fieten and her colleagues at the University of Utrecht identified the Labrador retriever with copper toxicosis as a canine model of Wilson disease,^35,39,40^ and a collaboration of the Linder laboratory and that of Hille Fieten found large quantities of low-molecular-weight Cu components in the blood plasma of Labrador retrievers, as shown in this paper. The Lutsenko laboratory thus provided the Linder laboratory with samples of blood plasma from the Atp7b−/− mice and found them to be rich in SCCs as well (also reported here). This led to the idea that in Cu overload, SCCs are secreted into the blood (probably by the liver), which filters them into urine, thus providing an alternative means of excreting the excess Cu. Indeed, Gray *et al.*’s data indicated that when urinary Cu levels began to rise, there was a concomitant decrease in liver Cu concentrations, although they still remained very high.^38^

Gray *et al.* had begun to purify the SCC but did not get very far.^38^ The Linder laboratory has been continuing those efforts and has made some progress. At the same time, investigations of the potential functions of SCC have begun, by producing ^67^Cu-labeled SCC in cultured cells and then feeding it to other cells. The results of the latter studies are also reported here.

## Materials and methods

### Blood plasma and urine samples

Heparinized blood plasma from Wilson disease (Atp7b−/−) model mice (on a C57BL6 background) was kindly provided by Abigael Muchenditsi, in the laboratory of Svetlana Lutsenko (Department of Physiology, Johns Hopkins Medical Institute, Baltimore, MD), and that of wild-type (C57BL6) mice by Christopher Vulpe and Brie Fuqua (Department of Nutrition and Toxicology, University of California Berkeley, Berkeley, CA), under IACUC protocols approved by the respective universities. Blood and urine samples from Labrador retriever dogs, with and without mutations in Atp7b, were collected in the clinic of the Department of Clinical Sciences of Companion Animals, University of Utrecht, under a protocol approved by the university IACUC. Blood plasma was obtained from heparinized whole blood collected from healthy adult human volunteers at the Student Health Center of California State University, Fullerton, under IRB-approved protocol HSR17-18-645 for Maria Linder.

### Cultured cells and treatments

Cells for the studies were rat hepatoma cells (RHCs; H-4-II-E), Caco2 cells [ATCC^®^ (HTB-37^TM^)], rat normal kidney endothelial cells (NRK-52E), and mouse embryonic fibroblasts (HDFa). Except for the fibroblasts, all were obtained directly from American Type Culture Collection (Manassas, VA). The fibroblasts—wild type and with copper transporter 1 knocked out (Ctr1−/−)—were kindly provided by Dennis Thiele (Duke University). All cells were maintained in Minimal Essential Medium (MEM, Gibco), supplemented with 10% fetal calf serum and 1.7 mM Na pyruvate and nonessential amino acids (Gibco). For experimental purposes, cells were grown in flasks ranging from T25 to T75 and T150, or in in six-well plates. To collect secretions, cells were either pretreated with 1–10 μM Cu(II) as the 1:1 nitrilotriacetate (Cu-NTA) complex or traces of radioactive ^67^Cu-NTA for 24 h, in maintenance culture medium, and then washed three times with MEM and reincubated in just MEM [not containing fetal bovine serum (FBS), etc.] for 24–48 h. The conditioned medium was collected for further studies and stored frozen at −20°C. Radioactive ^67^Cu was obtained from the Idaho State University Accelerator in Pocatello, ID. To produce a radiolabeled SCC, doses of ∼10 μCi were administered to the cells in T75 flasks containing 15 ml of culture medium, prior to collection of secretions.

### Partial purification of the small copper component(s)/carrier (SCC)

SCC (<1 kDa) was partially purified by various procedures that separated it from other molecules based on size and shape. The first step was usually ultrafiltration through filter units with a 10 kDa and then a 3 kDa cutoff, or just the latter (MilliporeSigma^TM^ Amicon^TM^ Ultra). Volumes of plasma or urine filtered were usually 2 ml, and half the volume of the filtrate was collected. For secreted SCC, volumes were either 2 or 15–30 ml, and 95% was filtered. Dialysis was with tubing that had a 0.1–0.5 kDa cutoff (Biotech CE dialysis tubing, Repligen). SCC samples were concentrated by freeze drying, with >90% recovery. Size exclusion chromatography (SEC) was carried out with 24 ml columns of Superdex 30 or 30 Increase (GE Healthcare; separation range 100–7000 Da) in a BioRad DuoFlow FPLC system, or in 100 ml gravity-flow columns of Biogel P4 (separation range 800–4000 Da; BioRad). For FPLC, 200 μl of sample was applied, and 0.5 ml fractions were collected. For the Biogel P4 columns, 1 ml of sample was applied, and 1 ml fractions were collected. SEC was carried out mostly with nanopure water and sometimes with 20 mM K phosphate, pH 7.0. Recoveries were >90%.

### Measuring rates of uptake of Cu from SCC and Cu-NTA

For this, cells were grown in six-well plates to ∼80% confluency and then incubated with ^67^Cu-labeled SCC-Cu or Cu-NTA. The ^67^Cu-SCC produced in rat hepatoma cells as described in the Cultured Cells section, was 3 kDa ultrafiltered, giving one radioactive Cu peak in SEC. ^67^Cu-labeled Cu-NTA was prepared by doping nonradioactive Cu(II)-NTA stock (equimolar Cu:NTA) with microliter quantities of radiotracer. Uptake was measured by following the accumulation of ^67^Cu within cells over periods ranging from 0.5 to 5 h (which was linear), but most studies with SCC were done using the 2 h time point. Radioactivity was counted in cells washed with MEM and lysed with lysis buffer [50 mM tris-HCl (pH 7.4), 150 mM NaCl, 1% Triton X-100, and 5 mM EDTA], using a Packard Cobra Quantum 5002 Gamma Counter. Uptake rates were calculated and are presented as percentage of dose per hour per milligram cell protein, after analysis of the lysates by BCA or Bradford assay, using bovine serum albumin as the standard. In some cases, cells were exposed to various concentrations of Ag(I) (nitrate), Cu(I) (chloride), Cu(II) (chloride), Ni(II) (nitrate), or Mn(II) (chloride) during uptake, or were preincubated for 30 min with endocytosis inhibitors—and then again during uptake. The endocytosis inhibitors [nocodazole, Dynamin Inhibitor I (Dynasore), and Pitstop^®^ 2] were given as a cocktail containing all three, each at final concentrations of 5 μM.

### Copper analysis

Cu was quantitated with a graphite furnace atomic absorption spectrometer (PerkinElmer AAnalyst^TM^ 600), with copper standards (SPEX CertiPrep^®^) diluted in 2% trace element grade nitric acid.

### Statistics

Data are presented as mean ± SD (*N*). Statistical analysis was performed by Student's *t*-test with unequal variance. *P*-values ≤0.05 were considered statistically significant.

## Results

### Elevation of small copper components in blood plasma and urine of animal models of Wilson disease.

**Fig. 1:**
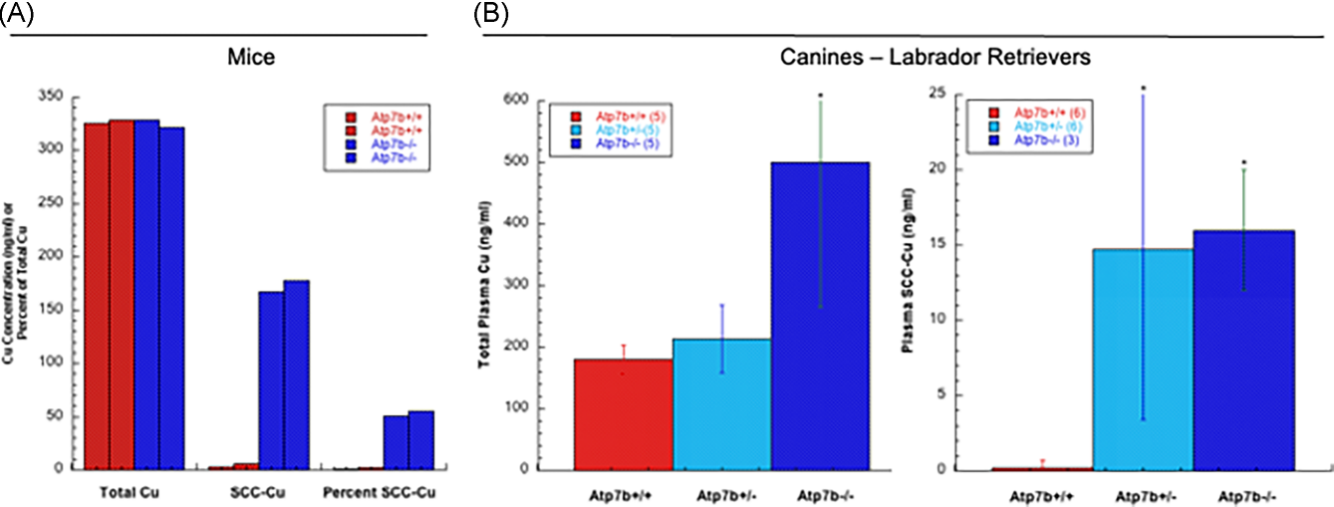
Proportions of blood plasma Cu associated with SCCs in mouse and canine models of Wilson disease (WD). (A) Concentrations of Cu (ng/ml) in whole plasma (total Cu) and 3 kDa filtrates (SCC-Cu) in samples from two wild-type (Atp7b+/+) and two WD model mice (Atp7b−/−) shown as individual red and blue bars, respectively, and percentage of total plasma Cu in SCC. (B) Total plasma Cu (left graph) and that of 3 kDa filtrates (SCC-Cu) (right graph) for Labrador retrievers that had normal Atp7b (Atp7b+/+, red) or were hetero- or homozygous for dysfunctional Atp7b (Atp7b+/+ and Atp7b−/− in light blue and dark blue, respectively). Data are means ± SD, for the number of samples indicated in parentheses in the figures. *P < 0.001 for difference from the wild type.

**Fig. 2:**
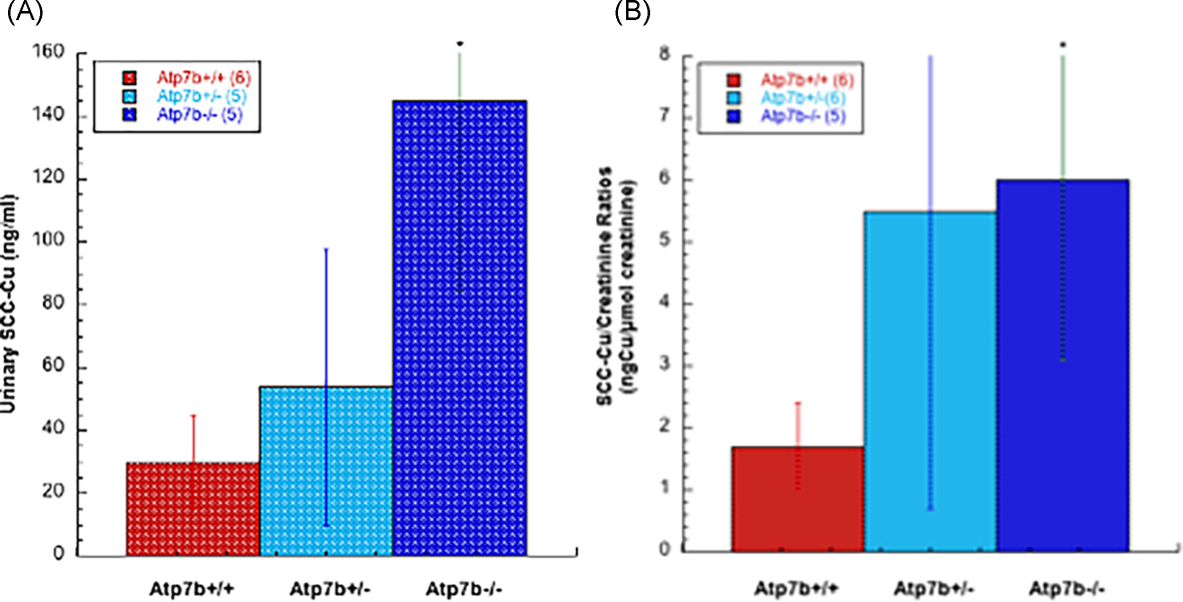
Levels of urinary copper associated with SCC in Labrador retriever models of WD. Ultrafiltrates (3 kDa cutoff) of urine samples from wild-type (WT, red) or hetero- (Atp7b-/+, light blue) and homozygote (Atp7b−/−, dark blue) animals were tested for Cu (designated SCC-Cu), and shown as nanograms of Cu per milliliter (A), or nanograms of Cu per micromole creatinine (B). Data are means ± SD for the numbers of samples indicated in parentheses. *P < 0.01–0.001 for difference from the wild type.

In the mouse model of Wilson disease, as in humans, Gray *et al.* demonstrated that large amounts of excess Cu accumulate when the Cu “pump” ATP7B is defective in hepatocytes.^38^ This was accompanied by a large increase in urinary Cu excretion (normally very low) in the form of a small molecule (less than 3 kDa).^38^ As shown below, blood plasma samples from two of the same Wilson disease model mice were then analyzed for total Cu as well as Cu in 3 kDa ultrafiltrates (Fig. 1A). While total plasma Cu levels were similar, those in the plasma ultrafiltrates of the wild type (containing SCC) were very low, whereas those from the Atp7b−/− mice were markedly elevated, accounting for about half of the total Cu present in the plasma. Blood plasma and urine samples for Labrador retriever dogs were also analyzed. The dogs were wild type, hetero, or homozygous with respect to Atp7b. Fig. 1B (left) shows the total plasma Cu values, which were elevated in the homozygous animals (Atp7b−/−). The right graph of Fig. 1B shows that wild-type retrievers (Atp7b+/+) had very little Cu (<1 ng/ml) in the 3 kDa ultrafiltrates of blood plasma (corresponding to SCC) but that there was a huge increase (to ∼15 ng/ml) in the case of both the homozygous and heterozygous canines with defective Atp7b. Urinary Cu excretion increased in parallel (Fig. 2). Although in the wild type there was a much higher concentration of Cu in the 3 kDa ultrafiltrate of urine (Fig. 2A) compared with blood plasma (Fig. 1B), there was a marked increase in the Cu content of the urinary 3 kDa ultrafiltrates of the homozygous animals (to ∼140 ng/ml), whether calculated as nanogram Cu per milliliter or relative to creatinine (Fig. 2A and B, respectively). For the heterozygotes, the increase in urinary ultrafiltrate Cu was much greater (and more similar to the changes in blood plasma ultrafiltrate) when calculated relative to concentrations of creatinine (Fig. 2B). (The latter is considered the most reliable measure, as it corrects for differences in daily urinary volume outputs, creatinine outputs being very constant, and its concentrations thus reflecting variations in dilution.) Three kDa ultrafiltrates of samples of blood plasma from six normal men and women taken two to four times over one year were also analyzed and showed very low levels of Cu (mostly around 4 ng Cu/ml, but two a bit higher). Together, the findings for the murine and canine models of Wilson disease imply that there is increased release of small copper component(s) into the blood fluid in the case of this Cu overload disease, and that this results in these components being filtered into urine, providing an alternative mechanism to excrete the accumulating Cu.

**Fig. 3:**
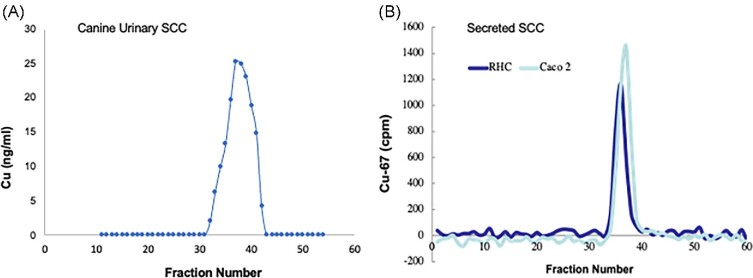
Approximate size of urinary and cell culture-secreted SCC, based on small-pore SEC on Superdex 30 (separation range 0.1–7 kDa). (A) Pooled 3 kDa ultrafiltrates of urine samples from the Labrador retrievers used in the studies shown in Fig. 2 were concentrated and applied to SEC, the fractions (0.5 ml) then being analyzed for Cu (ng/ml). (B) Here, 3 kDa ultrafiltrates of conditioned medium obtained from the secretions of rat hepatoma (RHC) and Caco2 (enterocyte model) cells that had been preloaded with traces of radioactive copper (^67^Cu) were applied to SEC, and the radioactivity of the eluted fractions was measured (cpm). Standards eluting on the same column were vitamin B12 (1350 Da) peaking at about fraction 35, and NADH (670 Da) peaking at about fraction 42.5.

To gauge the approximate size and potential heterogeneity of the urinary SCC, portions of the urinary ultrafiltrates remaining from the studies reported in Fig. 2 were pooled, concentrated, and applied to a small pore size exclusion column (Superdex 30). Fig. 3A shows that the Cu in the 3 kDa ultrafiltrate of the canine urine eluted as a somewhat broad band that peaked at about fraction 38. Since vitamin B12 (1350 Da) eluted at about fraction 35, and NADH (670 Da) at about fraction 42.5, it seems that the SCC is in the range of perhaps 700–1000 Da.

### Secretion of small copper components by cultured hepatic and intestinal cells

Since hepatocytes are the site for accumulation of excess Cu in Wilson disease and the cells most involved in maintaining homeostasis by excreting excess Cu into the bile, we wondered whether they might also be the ones producing the small Cu components/carriers (SCCs) detected in the plasma and urine in Wilson disease models. We thus cultured rat hepatoma cells (RHCs) in a medium containing traces of radioactive ^67^Cu(II)—given as the nitrilotriacetate complex (Cu-NTA) for 24 h—then washed away the tracer and incubated the cells for an additional 24 h in MEM (not containing FBS) to collect the secretions. As intestinal mucosal cells might conceivably also be a source of the SCC, we did the same with the Caco2 intestinal cell model. The resulting conditioned media were 3kDa ultrafiltered, and the ultrafiltrate applied to small-pore SEC on a Superdex 30 column. Fig. 3B shows the resulting elution profiles for ^67^Cu components. The conditioned medium of both cell types contained a single large peak of radioactive Cu, the elution of which peaked at fractions 36–37. The secreted Cu containing carriers eluted in about the same positions as the urinary SCC from Labrador retrievers (Fig. 3A), although the elution range was more narrow, suggesting one SCC component.

**Fig. 4:**
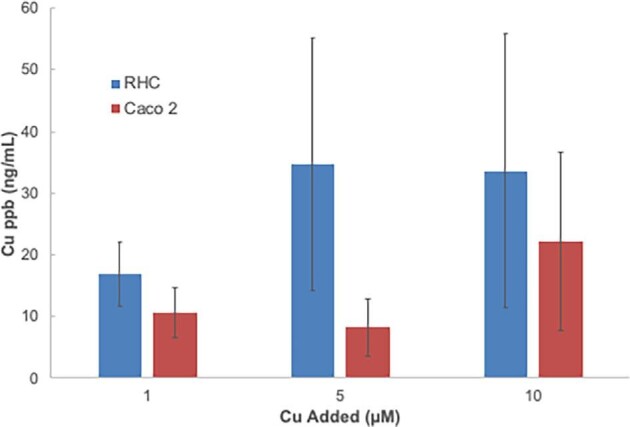
Amounts of SCC-Cu secreted by cultured hepatic and intestinal cells in response to preloading with added copper. Cells were preincubated for two days with tissue culture medium to which 1, 5, or 10 μM additional copper had been added in the form of Cu(II)-NTA. Cells were washed three times and further incubated for 24 h in just MEM, which had a starting Cu concentration of 0.02 μM. The conditioned medium was collected, ultrafiltered (3 kDa cutoff), and the concentration of Cu present was determined (ppb; ng/ml). Data for RHC (blue) and Caco2 cells (red) are given as means ± SD, for three separate batches of cells in each case. Differences due to dose were not statistically significant.

To determine whether Cu treatment would increase the secretion of the SCC, further studies were done with the RHC and Caco2 cells, collecting the conditioned medium after preincubating them with various concentrations of added Cu (1, 5, and 10 μM) given as non-radioactive Cu-NTA instead of radioactive copper tracer. The basal medium contained 0.02 μM Cu. Fig. 4 shows that preincubating cells with additional Cu-NTA increased the amounts of Cu collected in the 3 kDa ultrafiltrate of the conditioned medium enormously (>800-fold). This implies that excess Cu is stimulating SCC production and secretion. These data also showed that at least under conditions with excess Cu, the hepatic cells secreted more SCC than did those modeling the intestinal absorptive cells (Caco2).

### Uptake of SCC-Cu by cultured cells

**Fig. 5:**
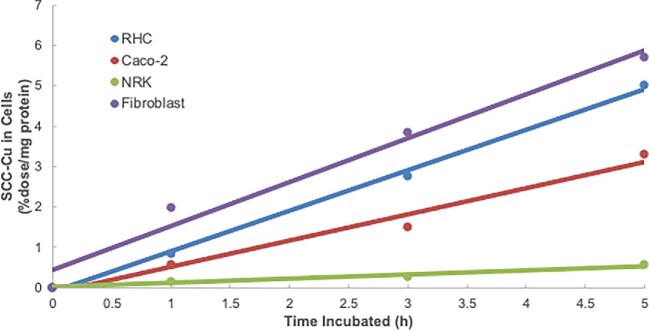
Cell uptake of ^67^Cu from secreted SCC by different cell types. Accumulation of ^67^Cu in hepatic (RHC), intestinal (Caco2), kidney epithelial (NRK), and fibroblast cells over time, when incubated with ^67^Cu-labeled SCC, produced by RHC cells. (For this, RHC cells were preloaded with radioactive Cu tracer, and then washed and incubated in MEM. The conditioned medium was 3 kDa ultrafiltered and the filtrate used as the source of ^67^Cu-SCC for the uptake studies.) Accumulation of radioactive Cu from SCC (as %dose/mg cell protein) is plotted against time (in h). RHC cell data are in blue; Caco2 cell data are in red; NRK cell data are in green; and fibroblast data are in purple. Each point on each line is the average of values for radioactivity in two different wells of washed cells. The best lines through these points have been drawn. The actual SCC-Cu concentration (after full radioactive decay) was 1.6 μM.

Since cells not being exposed to excess Cu seemed to secrete some SCC-Cu and yet very little (if any) of that showed up in the blood plasma and urine, we expected that the SCC-Cu secreted by hepatic or intestinal cells might be taken up by other cells. This idea was tested. Here, conditioned medium from RHC preloaded with traces of ^67^Cu-NTA was 3 kDa ultrafiltered, and the resulting medium (which showed a single large ^67^Cu peak in SEC; Fig. 3A) was incubated with various types of cultured cells. After 1, 3, and 5 h, cells were washed, lysed, and their radioactivity was determined. The results are shown in Fig. 5. All the cell types tested clearly took up Cu from the ^67^Cu-labeled SCC linearly, over time. Rates of uptake appeared similar in the case of all but the kidney epithelial cells (NRK), which were considerably lower. Notably, the two cell types that we already knew secreted SCC-Cu (RHC and Caco2) were also able to reabsorb it.

**Fig. 6:**
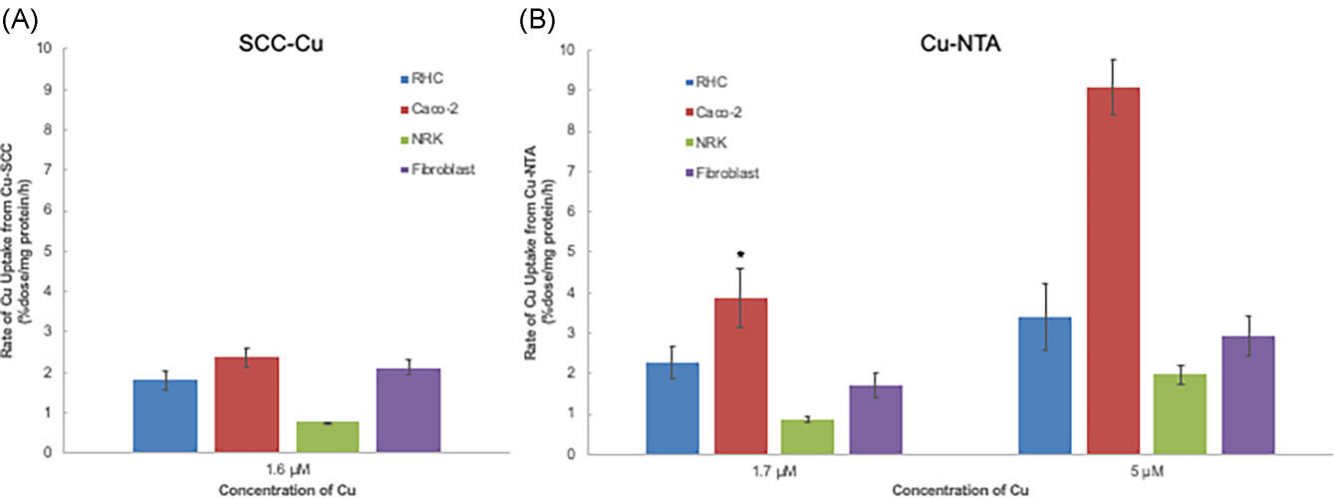
Relative rates of ^67^Cu uptake from SCC by different cell types (A), and comparison with rates of uptake of ionic copper given as Cu(II)-NTA (B). Rates of uptake of ^67^Cu were based on radioactivity accumulated over time in the various cell types, and are given as percentage of dose per milligram cell protein per hour, means ± SD. (A) The concentration of SCC-Cu was 1.6 μM and *N* = 4. (B) Concentrations of Cu(II)-NTA were 1.7 and 5.0 μM and *N* = 6, except for the NRK cells (*N* = 3). *P > 0.05 for difference from SCC-Cu uptake.

Based on the time course of these studies, it was decided that further uptake experiments would be based on measuring accumulation of ^67^Cu from SCC over the first 2 h and calculating rates as percentage of dose per milligram cell protein per hour. Results for the various cells are shown in Fig. 6A. These data confirmed that rates of uptake were similar for Caco2 cells, and fibroblasts, and RHCs in descending order, from ∼2.2% to ∼1.8% of the dose, but dropped to ∼0.5% in the case of NRK cells. The actual SCC-Cu concentration in these studies was 1.6 μM. We then compared these rates with those for uptake of ionic Cu, given as ^67^Cu-labeled Cu(II)-NTA (Fig. 6B). Uptake was measured at two different concentrations, 1.7 μM—similar to the 1.6 μM concentration for the SCC-Cu—and 5.0 μM, which is thought to be close to that for *V*_max_.^21^ At the similar/low concentration, relative rates of uptake from Cu(II)-NTA versus SCC-Cu were about the same for RHCs, fibroblasts, and NRK cells, but for the Caco2 cells the uptake rate from Cu-NTA was considerably faster. Increasing the Cu-NTA concentration to 5 μM increased uptake rates 30–50% in the RHCs and fibroblasts, and doubled rates in the case of the NRK and Caco2 cells. Thus, except perhaps for the Caco2 cells, SCC-Cu seemed to be about as readily available for uptake by the cells as the ionic Cu.

**Fig. 7:**
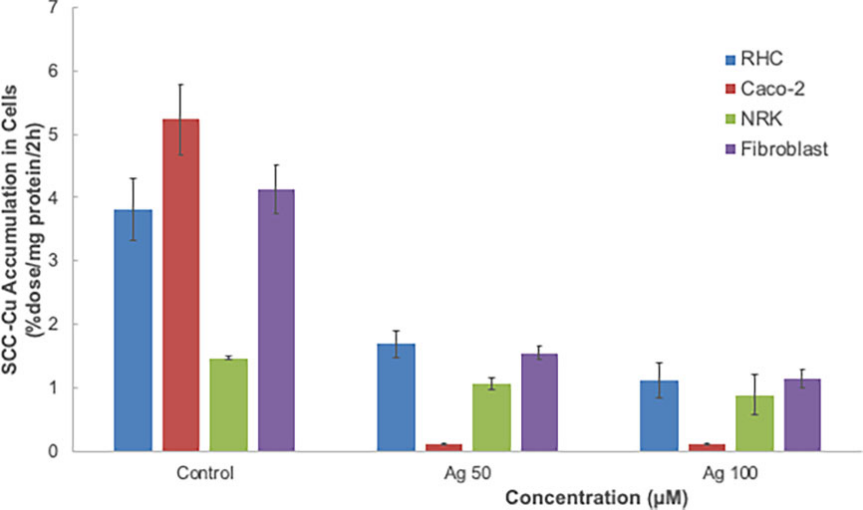
Relative rates of uptake of ^67^Cu from SCC by different cell types, and effects of excess silver ions. Rates of uptake of ^67^Cu from SCC secreted by RHC were measured based on radioactivity accumulated over 2 h in the various cell types (%dose/mg cell protein/2 h), in the absence (Control) and presence of an excess of 50 or 100 μM Ag(I). Data are means ± SD for *N* = 4. All effects of Ag(I) relative to controls were statistically significant: *P < 0.01–0.001.

The next question addressed was whether SCC-Cu was being taken up by CTR1, as had been suggested by some data obtained with the Wilson disease mouse model.^38^ We tested this in three different ways. First, it is well accepted that CTR1 takes up not only Cu(I) but also Ag(I), and that an excess of Ag(I) ions can thus inhibit Cu(I) uptake.^21,22,26,27^ So, we examined whether an excess of Ag(I) would inhibit uptake of ^67^Cu-labeled SCC-Cu. Fig. 7 shows that this was the case for three of the four cell types examined. A very large excess of Ag(I) (50 μM compared with 1.6 μM in the case of SCC-Cu) markedly inhibited ^67^Cu uptake from SCC by RHCs, Caco2 cells, and fibroblasts, and there was little additional inhibition with 100 μM Ag(I). In contrast, excess Ag(I) had relatively little effect on SCC-Cu uptake by NRK cells. Assuming that excess silver only inhibited Cu uptake by CTR1, these results imply not only that CTR1 takes up Cu from SCC but also that CTR1 expression varies widely among cell types. Thus, virtually all of the uptake of SCC-Cu by Caco2 cells was inhibited by excess Ag(I), but only ∼75% in the case of RHCs and fibroblasts, and only a tiny proportion in the case of the kidney epithelial (NRK) cells. The data also indicate and confirm that SCC-Cu can be absorbed not only by CTR1 but also by other transporters or processes.

**Fig. 8:**
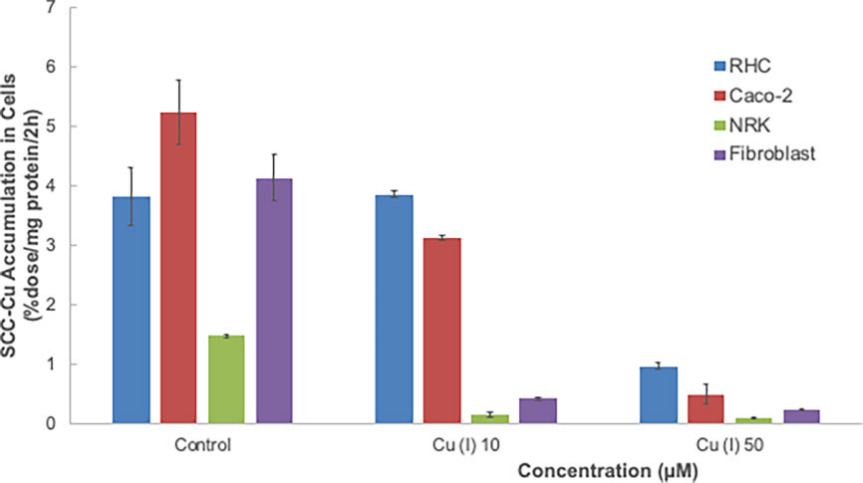
Effects of an excess of Cu(I) ions on rates of uptake of ^67^Cu from SCC by different cell types. Rates of uptake of ^67^Cu from SCC secreted by RHCs were measured based on radioactivity accumulated over 2 h in the various cell types (%dose/mg cell protein/2 h), in the absence (Control) and presence of an excess of 10 or 50 μM nonradioactive Cu(I). Data are means ± SD for *N* = 4. Except in the case of hepatic cells with 10 μM Cu(I), all effects of Cu(I) were statistically significant (*P* < 0.01–0.001).

Second, since CTR1 takes up Cu(I), just as with Ag(I) one would expect uptake of ^67^Cu from SCC to be inhibited by an excess of nonradioactive Cu(I) if CTR1 is involved. Fig. 8 shows that an excess of nonradioactive Cu(I) did markedly inhibit the uptake of ^67^Cu from SCC at the highest concentration tested (50 μM). At this concentration, inhibition by Cu(I) (Fig. 8) was greater than that produced by 50 uM Ag(I) (Fig. 7), ranging from 75% in the case of hepatic cells to 91% for Caco2 and kidney epithelial cells, and to 95% for fibroblasts. These results are again consistent with the involvement of CTR1, but also indicate that Cu(I) is interacting/inhibiting non-CTR1 uptake system(s) [not inhibited by Ag(I)] that are absorbing SCC-Cu. Uptake by NRK cells was almost completely inhibited by excess Cu(I) but hardly at all by excess Ag(I). Along the same lines, 10 (vs. 50) μM nonradioactive Cu(I) inhibited SCC-^67^Cu uptake by NRK cells and fibroblasts much more strongly than in the case of RHC and Caco2 cells. These anomalies suggest that the means by which Cu is absorbed from SCC involve several transporters or processes besides CTR1.

**Fig. 9:**
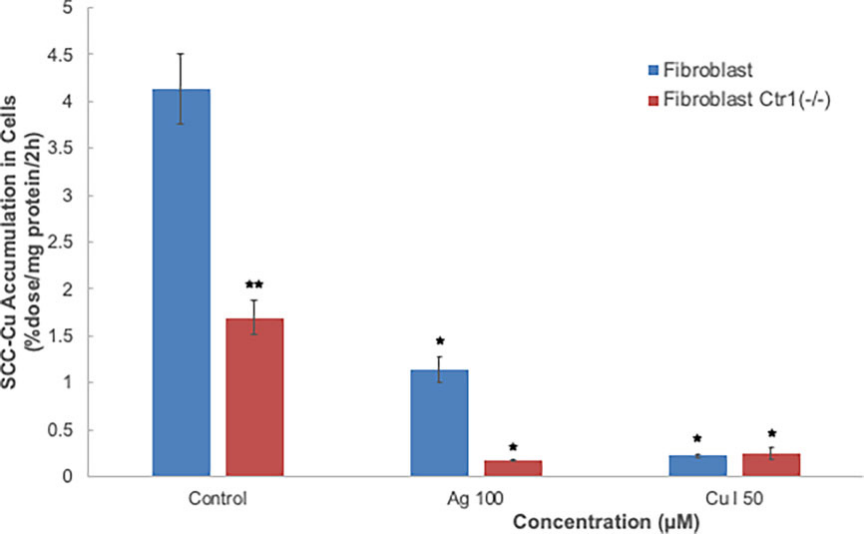
Effect of knocking out Ctr1 on uptake of ^67^Cu from SCC by mouse embryonic fibroblasts, plus responses to excess Ag(I) or Cu(I). Rates of uptake of ^67^Cu from SCC secreted by RHCs were measured based on radioactivity accumulated over 2 h (%dose/mg cell protein/2 h). Wild-type fibroblasts (blue) were compared with those where Ctr1 had been knocked out (Ctr1−/−, red). Data for effects of excess Ag(I) or Cu(I) (100 and 50 μM, respectively) on rates of uptake for Ctr1+/+ and −/− fibroblasts are also shown. Data are means ± SD for *N* = 4. **P < 0.001 for difference from wild type (Ctr1+/+); *P < 0.001 for effects of excess Ag(I) or Cu(I).

Finally, to prove that CTR1 was indeed involved, we compared rates of uptake in wild-type and Ctr1 knockout mouse embryonic fibroblasts. These were kindly provided by the laboratory of Dennis Thiele. The “Control” results in Fig. 9 show that knocking out this transporter reduced uptake rates by ∼60%. This proves that CTR1 can take up Cu complexed with SCC, and confirms that this uptake transporter is a major player in SCC-Cu uptake. As expected from the previous studies with wild-type fibroblasts, excess Cu(I) further inhibited the (already reduced) rate of uptake in the knockout cells (Fig. 9). Unexpectedly, however, excess Ag(I) also markedly further reduced SCC-Cu uptake in the knockout cells. Thus, while in the wild type knocking out CTR1 and treatment with 50 μM Ag(I) both reduced SCC-Cu uptake by 60% (Figs 7 and 9), 100 μM Ag(I) had additional effects in the knockout fibroblasts. This suggests that knocking out CTR1 might have been accompanied by an increased sensitivity to Ag(I), of unknown nature, causing toxicity.

**Fig. 10:**
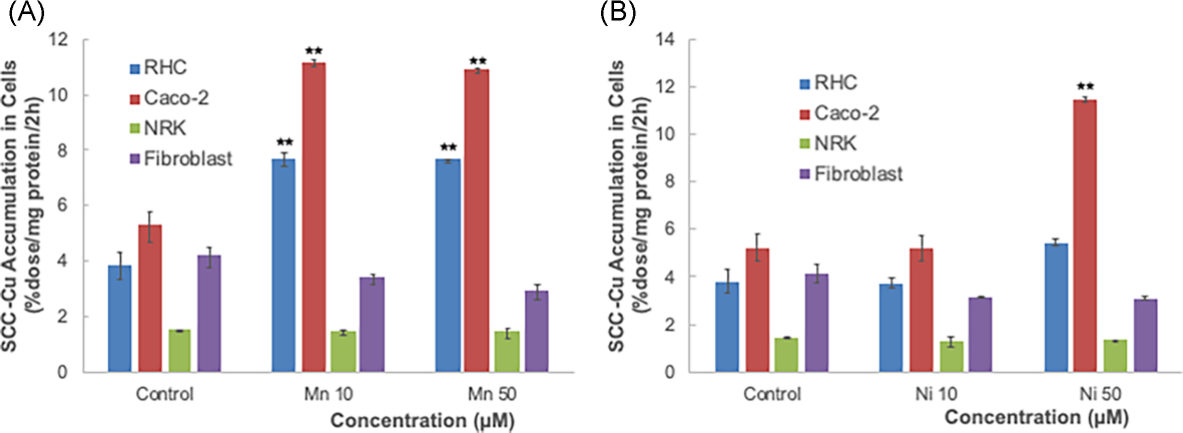
Effects of an excess of Ni(II) or Mn(II) ions that are substrates of DMT1 on uptake of ^67^Cu from SCC by different cell types. Rates of uptake of ^67^Cu from SCC secreted by RHCs were measured based on radioactivity accumulated over 2 h (%dose/mg cell protein/2 h), in the presence of high concentrations of Ni (A) or Mn (B). Data are means ± SD for *N* = 4. Only in the fibroblasts were small negative effects of Ni and Mn statistically significant (*P < 0.05–0.01). Some large positive effects were also statistically significant (**P < 0.001) but may reflect toxicity of those high metal concentrations.

Another transporter reputed to take Cu into cells is DMT1, although there has been continuing debate about whether or not this is actually the case.^29–33^ This transporter is the main way iron is absorbed from the digestive tract;^33,43^ and Cu(I) can inhibit this uptake, but not competitively.^44^ Several other metal ions, in their divalent forms, are taken up by DMT1,^33,41^ and include those of Zn, Ni, and Mn. To ascertain whether or not the Cu in SCC might be taken up by DMT1, we measured the uptake of ^67^Cu from radiolabeled SCC in the absence and presence of high concentrations of Ni(II) or Mn(II) ions (Fig. 10), which we would expect would compete with the Cu trying to enter via this transporter. In the case of all but the fibroblasts, no inhibition was observed. Indeed, in a few cases the rate of uptake was enhanced. (Why that would be the case is unknown, although toxicity cannot be ruled out.) For the fibroblasts, there was ∼25% inhibition of SCC-Cu uptake at the highest concentrations of Ni and Mn, suggesting a possible involvement of DMT1, but also perhaps a toxic effect. Overall, however, these data suggest that DMT1 is not important for uptake of Cu from SCC.

**Fig. 11:**
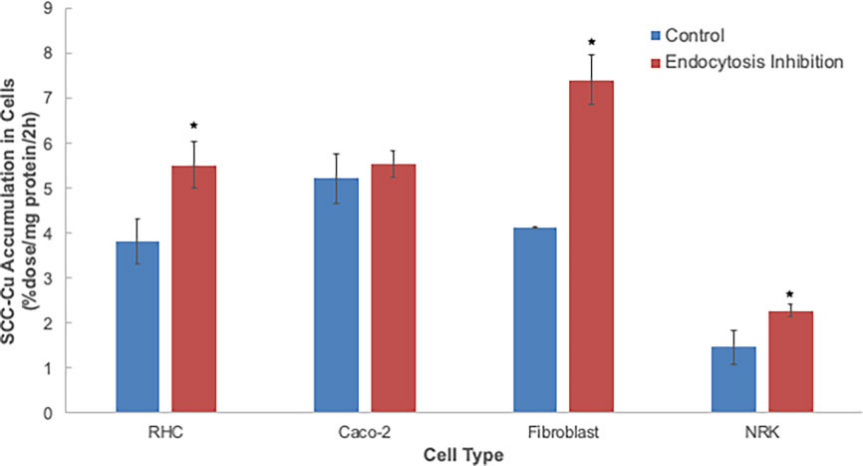
Effects of endocytosis inhibition on rates of uptake of ^67^Cu from SCC by various cell types. Rates of uptake of ^67^Cu from SCC secreted by RHCs were measured based on radioactivity accumulated over 2 h (%dose/mg cell protein/2 h). In each case, cells were not treated (blue) or treated (red) with a cocktail of three endocytosis inhibitors (Pitstop^®^2, Dynasore, and nocodazole), final concentrations of which were 5 μM. Treated cells were exposed to the inhibitors for 30 min prior to initiation of the uptake measurements as well as during the uptake period. Data are means ± SD for *N* = 4. *P < 0.01 for difference from untreated cells.

In the studies described, we were measuring accumulation of the radiolabeled Cu that was present in SCC. This does not tell us whether just the Cu in SCC or SCC as a whole (with its Cu) is being taken up. It is important to note that the Cu in SCC is bound very tightly, and cannot easily be removed *in vitro* even by reducing and chelating agents, except iminodiacetate (Miguel Tellez and Maria Linder, unpublished observations). SCC alone or its Cu complex (if both exist) is most likely too large to be taken up by CTR1 as a whole, or other typical metal uptake transporters, and the way most larger molecules enter cells is by endocytosis. We thus tested whether inhibitors of endocytosis would reduce rates of SCC-Cu uptake. The effect of a cocktail of three endocytosis inhibitors that target different aspects of the process was applied to determine whether uptake of ^67^Cu from SCC would be affected. The final concentrations of the inhibitors were in the lower part of the range normally used by others to inhibit endocytosis.^45–47^ The results are shown in Fig. 11. For none of the cell types was uptake of the Cu in SCC inhibited, strongly implying that the Cu in SCC, rather than SCC-Cu as a whole, is being absorbed by the cells.

## Discussion

In the studies reported here, we have obtained evidence that an SCC, less than 1 kDa in size, is secreted by some kinds of cells, and that secretion of this component is enhanced by pretreatment with high levels of Cu. The SCC produced in the normal state (without excess Cu) by the cultured cells elutes as a narrow band in small pore size exclusion chromatography, suggesting it is a single component. This is consistent with the results for a small Cu component present in the urine of WD model mice, reported by Gray *et al*.^38^ On the other hand, the SCC detected in the urine of Labrador retrievers eluted as a broader band, suggesting heterogeneity. Ongoing studies in the Linder laboratory have detected a similarly more broadly eluting component in porcine blood plasma that is being purified in larger quantities for the purpose of determining the actual chemical structure.

Our work shows that cultured cells modeling hepatocytes and enterocytes are secreting SCC, and that in both cell types, excess Cu enhances secretion. The secretory response to excess Cu of the hepatic cells is consistent with the fact that the liver is the main organ responsible for Cu homeostasis, and consistent with observations that accumulation of excess Cu in the livers of WD mouse models results in increased levels of SCC in the blood (reported here) and the urine.^38^ The same relationship between elevated SCC in plasma and urine has now been observed in the canine model of WD, which was first identified by Fieten *et al*.^39,40^ These findings corroborate and extend the concept first proposed by the Lutsenko laboratory that production/secretion of SCC provides an alternative route for excretion of excess Cu from the organism when biliary copper excretion is compromised and results in a buildup of liver Cu.^38^ The secretion of SCC by the enterocytic (Caco2) cell model implies that transfer of some of the Cu absorbed from the diet by enterocytes might be secreted into the blood as SCC rather than as ionic Cu, which is presumed to be released into the blood via ATP7A.^8^ However, at this point that is mere speculation. Indeed, we do not know whether the SCC released from the Caco2 cells might instead be entering the gut lumen. Whether other types of cells secrete SCC also remains to be explored.

Our findings that SCC-Cu is not only secreted but also absorbed by cells—including those that secrete it—adds a new dimension to the concepts of how Cu is distributed in the mammalian organism to maintain Cu homeostasis. The current “dogma” is that Cu is absorbed from the diet in ionic form (via CTR1 and other transporters), and that this Cu is transferred to the “Cu pump,” ATP7A, in the TGN (to enter vesicles for exocytosis) and/or at the basolateral cell surface, for release into the interstitial fluid and blood circulation. There it binds proteins that have high affinities for Cu(II) (including albumin) in the portal vein and delivers most of the Cu to the liver (and also the kidney).^1,2,4,48^ Complexes of these proteins with radiolabeled ionic Cu deliver it to cells—as shown in cell culture studies—where it is taken up by CTR1 as well as by at least one other Cu uptake transporter.^21,22^ Uptake of Cu(II) carried by plasma proteins is thought to be mediated by cell surface reductases (dCytB, Steap proteins)^41,42^ that also reduce Fe(III), since Cu(II) uptake is inhibited by excess Fe(III).^9,21,22^ The results presented here suggest that in addition to the current dogma described, Cu is being secreted into the blood as a small stable molecule (SCC) at least by some cells, under normal conditions, and that the Cu in SCC is then taken up by neighboring cells in the same organ or other organs, showing a new way by which Cu can be distributed around the organism in support of Cu homeostasis. Secretion of SCC might thus be another way of providing Cu to cells that need it as well as a means of releasing excess Cu. Moreover, when SCC-Cu levels get too high in the blood fluid, the kidney would not only filter the SCC-Cu into the tubules, but perhaps would also decrease rates of SCC-Cu reabsorption so that the excess SCC-Cu ends up in the urine. We do not think it likely that the Cu and SCC that binds it are separately released from the cells, because first of all, small-pore SEC does not show a Cu complex such as Cu(II)-di-histidine (that might have been formed between ionic Cu released by ATP7A, e.g.) and histidine (100 μM) in the culture medium—which contains no plasma proteins that would otherwise bind it. Second, the SCC-Cu complex is very stable, not releasing its Cu when extensively dialyzed against buffers ranging from pH 4 to 8, in the presence of 100 μM imidazole (Sandy Ma and Maria Linder, unpublished).

Our finding that the Cu in SCC is taken up by cells expands on the initial findings reported by Gray et al.^38^ that SCC inhibits uptake of ionic Cu by cells, suggesting it competes for uptake. Use of low concentrations of Cu-radiolabeled SCC allowed us to show that unlabeled Cu(I) was indeed competing with the SCC-Cu for uptake. Moreover, an excess of Ag(I) ions also competed, implying that CTR1 might be involved. This was also proposed by Gray *et al.*,^38^ who found that expression of this Cu(I) [and Ag(I)] uptake transporter was downregulated in the livers of the WD model mice—who had high levels of urinary SCC,^38^ as well as high levels of plasma SCC (reported here). We have confirmed and established that CTR1 is indeed able to take up Cu from SCC. Fibroblasts where the transporter had been knocked out took up SCC-Cu at a 60% lower rate than the wild type, indicating that in this cell type, 60% of the SCC-Cu uptake is normally via CTR1. This agrees with the 61% inhibition of Cu uptake induced by 50 μM Ag(I). In the intestinal cells, addition of excess Ag(I) completely inhibited uptake of SCC-^67^Cu, implying that here it might be the only transporter involved in SCC-Cu uptake. On the other end of the spectrum, uptake of SCC-Cu by the kidney epithelial cells was only mildly inhibited by excess Ag(I), indicating that as in fibroblasts another uptake system is also present. In hepatic cells and fibroblasts, CTR1 accounted for about two-thirds of the uptake—based on the degree of Ag(I) inhibition. Nonradioactive Cu(I) competed for uptake with SCC-Cu in every cell type, but almost completely blocked its uptake only in the kidney epithelial cells. These cells had the lowest rates of SCC (and ionic) Cu uptake, and very little CTR1; that is, there was almost no inhibition by Ag(I). This uptake, by some unknown transporter, was inhibited by an excess of Cu(I), implying that this is the form in which Cu is taken up by this transporter. In none of the cell types was uptake of the Cu from SCC reduced by inhibitors of endocytosis. This implies that Cu from SCC is entering as the ion and not as the SCC-Cu complex, and is also consistent with the concept that ionic Cu and Ag are competing with SCC-Cu for uptake.

Since there are reports that at least in some conditions, DMT1 may also be taking up Cu,^29–31^ although other data imply this is not the case,^33,33^ we examined for potentially inhibitory effects on SCC-Cu uptake by high concentrations of metal ions that are known substrates of DMT1. A small inhibitory effect (∼20% reduction) on the rate of uptake was observed in the case of the fibroblasts, but no inhibition at all in the case of the other three cell types. This implies that DMT1 is very unlikely to be a major player in uptake of Cu from SCC.

In conclusion, it seems that SCC is a normal component of the blood circulation. Although normally present in very low concentrations, small but variable amounts of Cu components <3 kDa in size are clearly present, and the values appear to differ among species. For example, the SCC-Cu content of pig plasma that we are using as a source of SCC is relatively high but also quite variable (in the range from 30 to 100 ng Cu/ml), and there is also variability in the few samples of plasma from normal humans we tested here (from 4 to ∼90 ng Cu/ml), although most are at the low end. Clearly, Cu overload results in much higher levels, which are likely to be due to the excess Cu accumulating in the liver and perhaps also in other cell types that then secrete it. Blood fluid levels of SCC are most likely determined by relative rates of secretion into and uptake from the blood plasma. Some investigators have reported on levels of what is most probably SCC-Cu in Alzheimer's disease,^49,50^ using an assay for “free” or “non-ceruloplasmin” Cu developed by Squitti and colleagues.^49^ (Some assays for “free” or “non-ceruloplasmin” Cu use a different approach that is not measuring SCC-Cu.^51,53^) Thus, SCC levels may be related to physiological conditions other than Cu overload, or reflect other perturbations of the homeostasis of this metal.

## Data Availability

All data are incorporated in the article.
